# Dibromido{2-[(4-fluoro­phen­yl)imino­meth­yl]pyridine-κ^2^ *N*,*N*′}zinc

**DOI:** 10.1107/S1600536812031091

**Published:** 2012-07-25

**Authors:** Saeed Dehghanpour, Ali Mahmoudi

**Affiliations:** a Department of Chemistry, Islamic Azad University, Karaj, Iran

## Abstract

In the title complex, [ZnBr_2_(C_12_H_9_FN_2_)], the Zn^II^ atom has a distorted tetra­hedral Br_2_N_2_ coordination sphere. The organic ligand is bidentate, coordinating the Zn^II^ atom *via* two imine N atoms. The benzene and pyridine rings are oriented at a dihedral angle of 10.49 (1)°. In the crystal, weak C—H⋯F and C—H⋯Br hydrogen bonds are observed.

## Related literature

For background information, see: Dehghanpour *et al.* (2009 ▶). For related structures, see: Dehghanpour *et al.* (2007 ▶); Salehzadeh *et al.* (2011 ▶); Khalaj *et al.* (2009 ▶).
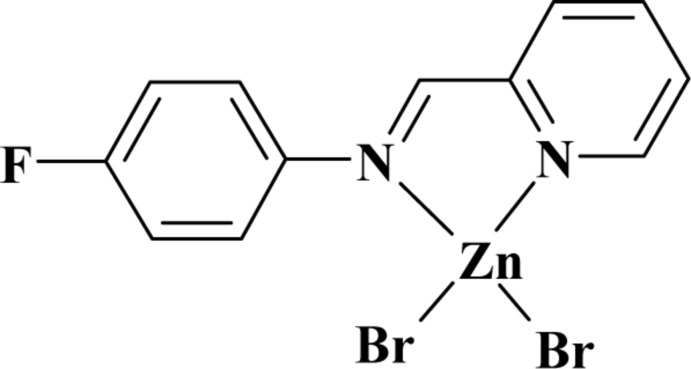


## Experimental

### 

#### Crystal data

[ZnBr_2_(C_12_H_9_FN_2_)]
*M* *_r_* = 425.40
Monoclinic, 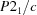

*a* = 7.7351 (10) Å
*b* = 9.5372 (13) Å
*c* = 18.501 (2) Å
β = 96.052 (3)°
*V* = 1357.2 (3) Å^3^
*Z* = 4
Mo *K*α radiation
μ = 7.69 mm^−1^
*T* = 100 K
0.17 × 0.06 × 0.04 mm

#### Data collection

Bruker APEXII area-detector diffractometer
Absorption correction: multi-scan (*APEX2*; Bruker, 2005 ▶) *T*_min_ = 0.435, *T*_max_ = 0.734
18332 measured reflections
2956 independent reflections
2469 reflections with *I* > 2σ(*I*)
*R*_int_ = 0.049

#### Refinement

*R*[*F* ^2^ > 2σ(*F* ^2^)] = 0.030
*wR*(*F* ^2^) = 0.072
*S* = 1.00
2956 reflections
163 parameters
H-atom parameters constrained
Δρ_max_ = 2.44 e Å^−3^
Δρ_min_ = −0.77 e Å^−3^

### 

Data collection: *APEX2* (Bruker, 2005 ▶); cell refinement: *SAINT* (Bruker, 2005 ▶); data reduction: *SAINT*; program(s) used to solve structure: *SHELXS97* (Sheldrick, 2008 ▶); program(s) used to refine structure: *SHELXL97* (Sheldrick, 2008 ▶); molecular graphics: *SHELXTL* (Sheldrick, 2008 ▶); software used to prepare material for publication: *SHELXTL*.

## Supplementary Material

Crystal structure: contains datablock(s) I, global. DOI: 10.1107/S1600536812031091/pv2564sup1.cif

Structure factors: contains datablock(s) I. DOI: 10.1107/S1600536812031091/pv2564Isup2.hkl

Additional supplementary materials:  crystallographic information; 3D view; checkCIF report

## Figures and Tables

**Table 1:** Hydrogen-bond geometry (Å, °)

*D*—H⋯*A*	*D*—H	H⋯*A*	*D*⋯*A*	*D*—H⋯*A*
C12—H12*A*⋯Br1	0.93	3.04	3.866 (4)	148
C2—H2*A*⋯F1^i^	0.93	2.50	3.081 (4)	121
C5—H5*A*⋯Br1^ii^	0.93	3.01	3.767 (4)	140
C6—H6*A*⋯Br1^iii^	0.93	3.05	3.756 (4)	134
C3—H3*A*⋯Br2^iv^	0.93	2.90	3.810 (4)	166

Symmetry codes: (i) 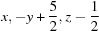; (ii) 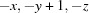; (iii) 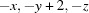; (iv) 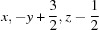.

**Fig. 1.**
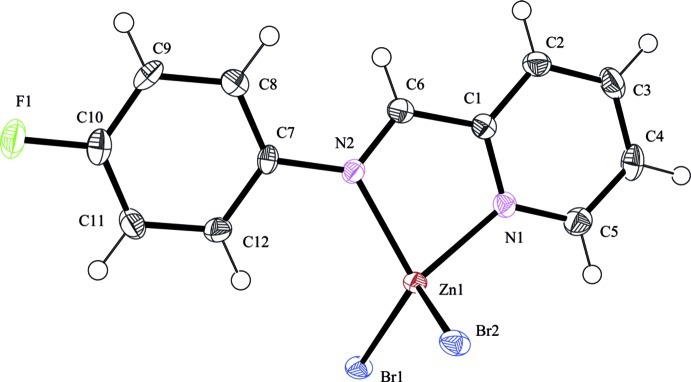
The molecular structure of the title compound with the atom numbering scheme. Displacement ellipsoids are drawn at the 50% probability level. H atoms are presented as small spheres of arbitrary radius.

**Fig. 2.**
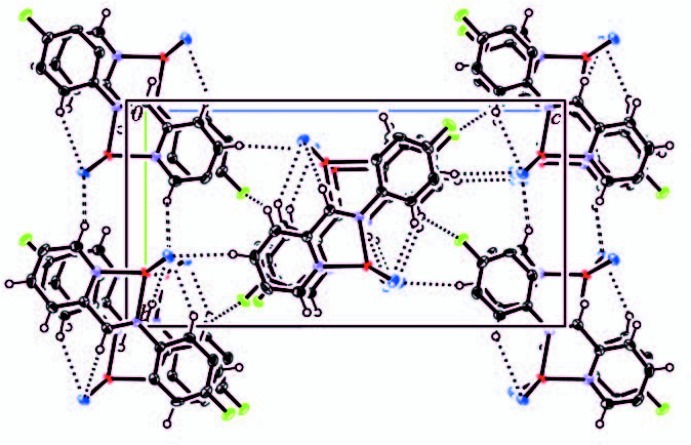
A view of the C—H···F and C—H···Br hydrogen bonds (dotted lines) in the crystal structure of the title compound. H atoms non-participating in hydrogen-bonding were omitted for clarity.

## supplementary crystallographic information

### Comment

In continuation of our research on the synthesis and characterization of metal complexes containing bidentate Schiff base ligands (Dehghanpour *et al.* (2009)), we now report the synthesis and crystal structure of a zinc complex of the Schiff base, (4-fluorophenyl)pyridin-2-ylmethyleneamine.

The metal centre in the title complex (Fig. 1) has a tetrahedral coordination which shows signficant distortion, mainly due to the presence of the five-membered chelate ring. The endocyclic N1—Zn1—N2 angle (81.13 (12)°) is much narrower than the ideal tetrahedral angle of 109.5°, whereas the opposite Br1—Zn1—Br2 angle (116.72 (2)°) is much wider than the ideal tetrahedral angle. The Zn—Br and Zn—N bond distances compare well with the values found in other tetrahedral Schiff base adducts of zinc bromide (Salehzadeh *et al.*, 2011; Dehghanpour *et al.*, 2007; Khalaj *et al.*, 2009). The interplanar angles between the benzene and pyridine rings in the title structure is 10.49 (1)°. In the crystal, weak C—H···F and C—H···Br hydrogen bonds are also observed (Tab. 1 & Fig. 2).

### Experimental

The title complex was prepared by the reaction of ZnBr_2_ (22.5 mg, 0.1 mmol) and (4-fluorophenyl)pyridin-2-ylmethyleneamine (20 mg, 0.1 mmol) in 10 ml of methanol at room temperature. The solution was allowed to stand at room temperature and crystals of the title compound suitable for X-ray analysis formed within a few days.

### Refinement

Though the H-atoms were observable in the difference electron density maps they were included at geometrically idealized positions with C—H distances = 0.93 Å and U_iso_ = 1.2 times U_eq_ of the atoms to which they were bonded. There is a high positive residual density of 2.44 e Å^-3^ near the Zn1 center due to considerable absorption effects which could not be completely corrected.

### Crystal data

**Table:**

[ZnBr_2_(C_12_H_9_FN_2_)]	*F*(000) = 816
*M**_r_* = 425.40	*D*_x_ = 2.082 Mg m^−^^3^
Monoclinic, *P*2_1_/*c*	Mo *K*α radiation, λ = 0.71073 Å
Hall symbol: -P 2ybc	Cell parameters from 3858 reflections
*a* = 7.7351 (10) Å	θ = 2.2–29.7°
*b* = 9.5372 (13) Å	µ = 7.69 mm^−^^1^
*c* = 18.501 (2) Å	*T* = 100 K
β = 96.052 (3)°	Prism, colourless
*V* = 1357.2 (3) Å^3^	0.17 × 0.06 × 0.04 mm
*Z* = 4	

### Data collection

**Table:**

Bruker APEXII area-detector diffractometer	2956 independent reflections
Radiation source: fine-focus sealed tube	2469 reflections with *I* > 2σ(*I*)
Graphite monochromator	*R*_int_ = 0.049
ω scans	θ_max_ = 27.0°, θ_min_ = 2.2°
Absorption correction: multi-scan (*APEX2*; Bruker, 2005)	*h* = −9→9
*T*_min_ = 0.435, *T*_max_ = 0.734	*k* = −12→12
18332 measured reflections	*l* = −23→23

### Refinement

**Table:**

Refinement on *F*^2^	Primary atom site location: structure-invariant direct methods
Least-squares matrix: full	Secondary atom site location: difference Fourier map
*R*[*F*^2^ > 2σ(*F*^2^)] = 0.030	Hydrogen site location: inferred from neighbouring sites
*wR*(*F*^2^) = 0.072	H-atom parameters constrained
*S* = 1.00	*w* = 1/[σ^2^(*F*_o_^2^) + (0.0358*P*)^2^ + 2.3621*P*] where *P* = (*F*_o_^2^ + 2*F*_c_^2^)/3
2956 reflections	(Δ/σ)_max_ = 0.001
163 parameters	Δρ_max_ = 2.44 e Å^−^^3^
0 restraints	Δρ_min_ = −0.77 e Å^−^^3^

### Special details

**Table:**

Geometry. All e.s.d.'s (except the e.s.d. in the dihedral angle between two l.s. planes) are estimated using the full covariance matrix. The cell e.s.d.'s are taken into account individually in the estimation of e.s.d.'s in distances, angles and torsion angles; correlations between e.s.d.'s in cell parameters are only used when they are defined by crystal symmetry. An approximate (isotropic) treatment of cell e.s.d.'s is used for estimating e.s.d.'s involving l.s. planes.
Refinement. Refinement of *F*^2^ against ALL reflections. The weighted *R*-factor *wR* and goodness of fit *S* are based on *F*^2^, conventional *R*-factors *R* are based on *F*, with *F* set to zero for negative *F*^2^. The threshold expression of *F*^2^ > σ(*F*^2^) is used only for calculating *R*-factors(gt) *etc*. and is not relevant to the choice of reflections for refinement. *R*-factors based on *F*^2^ are statistically about twice as large as those based on *F*, and *R*- factors based on ALL data will be even larger.

### Fractional atomic coordinates and isotropic or equivalent isotropic displacement parameters (Å^2^)

**Table:**

	*x*	*y*	*z*	*U*_iso_*/*U*_eq_	
Br1	−0.00870 (4)	0.67594 (4)	0.09661 (2)	0.01923 (10)	
Br2	0.50834 (4)	0.69666 (4)	0.11656 (2)	0.02040 (10)	
Zn1	0.24082 (5)	0.76510 (4)	0.05390 (2)	0.01606 (11)	
N2	0.2404 (4)	0.9803 (3)	0.03443 (16)	0.0159 (6)	
C1	0.2874 (4)	0.8917 (4)	−0.08143 (19)	0.0166 (7)	
F1	0.1273 (3)	1.3856 (3)	0.23727 (12)	0.0320 (6)	
C8	0.2592 (5)	1.2286 (4)	0.0745 (2)	0.0193 (8)	
H8A	0.3069	1.2546	0.0324	0.023*	
C9	0.2290 (5)	1.3290 (4)	0.1259 (2)	0.0227 (8)	
H9A	0.2547	1.4229	0.1187	0.027*	
C2	0.3253 (5)	0.9156 (4)	−0.1518 (2)	0.0223 (8)	
H2A	0.3411	1.0063	−0.1683	0.027*	
C10	0.1598 (5)	1.2862 (4)	0.1881 (2)	0.0232 (8)	
C6	0.2678 (4)	1.0072 (4)	−0.03110 (19)	0.0180 (7)	
H6A	0.2753	1.0996	−0.0466	0.022*	
C12	0.1525 (5)	1.0492 (4)	0.1500 (2)	0.0205 (8)	
H12A	0.1290	0.9552	0.1582	0.025*	
C4	0.3159 (5)	0.6678 (4)	−0.1709 (2)	0.0259 (9)	
H4A	0.3237	0.5900	−0.2006	0.031*	
C7	0.2183 (4)	1.0901 (4)	0.08600 (19)	0.0160 (7)	
C3	0.3393 (5)	0.8013 (5)	−0.1973 (2)	0.0264 (9)	
H3A	0.3642	0.8144	−0.2450	0.032*	
C5	0.2804 (5)	0.6514 (4)	−0.0995 (2)	0.0232 (8)	
H5A	0.2662	0.5615	−0.0817	0.028*	
C11	0.1222 (5)	1.1492 (4)	0.2015 (2)	0.0240 (8)	
H11A	0.0771	1.1237	0.2443	0.029*	
N1	0.2661 (4)	0.7613 (3)	−0.05549 (16)	0.0176 (6)	

### Atomic displacement parameters (Å^2^)

**Table:**

	*U*^11^	*U*^22^	*U*^33^	*U*^12^	*U*^13^	*U*^23^
Br1	0.01582 (18)	0.01869 (19)	0.0241 (2)	−0.00193 (13)	0.00645 (13)	0.00462 (15)
Br2	0.01554 (18)	0.0241 (2)	0.0222 (2)	0.00173 (14)	0.00489 (13)	0.00680 (15)
Zn1	0.0172 (2)	0.0141 (2)	0.0177 (2)	−0.00006 (16)	0.00594 (15)	0.00254 (16)
N2	0.0161 (15)	0.0135 (15)	0.0183 (15)	−0.0001 (12)	0.0029 (11)	−0.0010 (12)
C1	0.0130 (16)	0.0193 (19)	0.0175 (17)	0.0004 (14)	0.0011 (13)	0.0007 (15)
F1	0.0414 (14)	0.0251 (13)	0.0298 (13)	0.0036 (11)	0.0046 (10)	−0.0122 (10)
C8	0.0170 (17)	0.0229 (19)	0.0182 (18)	0.0014 (15)	0.0020 (14)	0.0022 (15)
C9	0.026 (2)	0.0112 (18)	0.030 (2)	0.0010 (14)	−0.0023 (16)	−0.0023 (16)
C2	0.0223 (19)	0.022 (2)	0.023 (2)	0.0031 (15)	0.0046 (15)	0.0059 (16)
C10	0.0210 (19)	0.025 (2)	0.0231 (19)	0.0029 (16)	−0.0008 (15)	−0.0089 (17)
C6	0.0142 (17)	0.0202 (19)	0.0198 (18)	0.0021 (14)	0.0026 (13)	0.0039 (15)
C12	0.0218 (19)	0.0165 (18)	0.0238 (19)	−0.0012 (15)	0.0058 (15)	0.0024 (15)
C4	0.028 (2)	0.027 (2)	0.023 (2)	0.0038 (17)	0.0005 (16)	−0.0125 (17)
C7	0.0113 (16)	0.0175 (18)	0.0192 (18)	0.0041 (13)	0.0016 (13)	−0.0037 (14)
C3	0.028 (2)	0.036 (2)	0.0157 (18)	0.0081 (18)	0.0056 (15)	0.0008 (17)
C5	0.0207 (19)	0.020 (2)	0.030 (2)	−0.0019 (15)	0.0052 (16)	−0.0001 (16)
C11	0.026 (2)	0.028 (2)	0.0185 (19)	0.0001 (16)	0.0077 (15)	−0.0019 (16)
N1	0.0155 (15)	0.0181 (16)	0.0198 (15)	0.0003 (12)	0.0045 (12)	−0.0007 (13)

### Geometric parameters (Å, º)

**Table:**

Br1—Zn1	2.3225 (6)	C2—C3	1.389 (6)
Br2—Zn1	2.3550 (6)	C2—H2A	0.9300
Zn1—N1	2.054 (3)	C10—C11	1.366 (6)
Zn1—N2	2.084 (3)	C6—H6A	0.9300
N2—C6	1.279 (5)	C12—C11	1.385 (5)
N2—C7	1.439 (4)	C12—C7	1.393 (5)
C1—N1	1.350 (5)	C12—H12A	0.9300
C1—C2	1.383 (5)	C4—C3	1.383 (6)
C1—C6	1.461 (5)	C4—C5	1.385 (6)
F1—C10	1.356 (4)	C4—H4A	0.9300
C8—C7	1.380 (5)	C3—H3A	0.9300
C8—C9	1.386 (5)	C5—N1	1.339 (5)
C8—H8A	0.9300	C5—H5A	0.9300
C9—C10	1.381 (6)	C11—H11A	0.9300
C9—H9A	0.9300		
			
N1—Zn1—N2	81.13 (12)	N2—C6—C1	119.4 (3)
N1—Zn1—Br1	119.84 (8)	N2—C6—H6A	120.3
N2—Zn1—Br1	115.66 (8)	C1—C6—H6A	120.3
N1—Zn1—Br2	108.07 (8)	C11—C12—C7	119.7 (4)
N2—Zn1—Br2	110.09 (8)	C11—C12—H12A	120.1
Br1—Zn1—Br2	116.72 (2)	C7—C12—H12A	120.1
C6—N2—C7	121.7 (3)	C3—C4—C5	119.2 (4)
C6—N2—Zn1	111.4 (3)	C3—C4—H4A	120.4
C7—N2—Zn1	126.9 (2)	C5—C4—H4A	120.4
N1—C1—C2	122.1 (3)	C8—C7—C12	120.6 (3)
N1—C1—C6	116.3 (3)	C8—C7—N2	123.3 (3)
C2—C1—C6	121.5 (3)	C12—C7—N2	116.1 (3)
C7—C8—C9	119.8 (3)	C4—C3—C2	119.1 (4)
C7—C8—H8A	120.1	C4—C3—H3A	120.5
C9—C8—H8A	120.1	C2—C3—H3A	120.5
C10—C9—C8	118.4 (4)	N1—C5—C4	121.9 (4)
C10—C9—H9A	120.8	N1—C5—H5A	119.0
C8—C9—H9A	120.8	C4—C5—H5A	119.0
C1—C2—C3	118.7 (4)	C10—C11—C12	118.6 (4)
C1—C2—H2A	120.7	C10—C11—H11A	120.7
C3—C2—H2A	120.7	C12—C11—H11A	120.7
F1—C10—C11	119.3 (4)	C5—N1—C1	118.9 (3)
F1—C10—C9	117.9 (3)	C5—N1—Zn1	129.5 (3)
C11—C10—C9	122.8 (4)	C1—N1—Zn1	111.2 (2)
			
N1—Zn1—N2—C6	−4.7 (3)	C6—N2—C7—C12	166.6 (3)
Br1—Zn1—N2—C6	−123.7 (2)	Zn1—N2—C7—C12	−15.4 (4)
Br2—Zn1—N2—C6	101.4 (2)	C5—C4—C3—C2	−0.6 (6)
N1—Zn1—N2—C7	177.1 (3)	C1—C2—C3—C4	−0.3 (6)
Br1—Zn1—N2—C7	58.2 (3)	C3—C4—C5—N1	0.8 (6)
Br2—Zn1—N2—C7	−76.8 (3)	F1—C10—C11—C12	178.6 (3)
C7—C8—C9—C10	0.7 (5)	C9—C10—C11—C12	−0.9 (6)
N1—C1—C2—C3	0.9 (5)	C7—C12—C11—C10	−0.7 (6)
C6—C1—C2—C3	−178.9 (3)	C4—C5—N1—C1	−0.2 (5)
C8—C9—C10—F1	−178.6 (3)	C4—C5—N1—Zn1	−172.7 (3)
C8—C9—C10—C11	0.9 (6)	C2—C1—N1—C5	−0.7 (5)
C7—N2—C6—C1	−179.3 (3)	C6—C1—N1—C5	179.2 (3)
Zn1—N2—C6—C1	2.4 (4)	C2—C1—N1—Zn1	173.1 (3)
N1—C1—C6—N2	3.2 (5)	C6—C1—N1—Zn1	−7.0 (4)
C2—C1—C6—N2	−176.9 (3)	N2—Zn1—N1—C5	179.3 (3)
C9—C8—C7—C12	−2.3 (5)	Br1—Zn1—N1—C5	−66.1 (3)
C9—C8—C7—N2	178.0 (3)	Br2—Zn1—N1—C5	71.0 (3)
C11—C12—C7—C8	2.3 (5)	N2—Zn1—N1—C1	6.3 (2)
C11—C12—C7—N2	−178.0 (3)	Br1—Zn1—N1—C1	120.9 (2)
C6—N2—C7—C8	−13.7 (5)	Br2—Zn1—N1—C1	−102.0 (2)
Zn1—N2—C7—C8	164.3 (3)		

### Hydrogen-bond geometry (Å, º)

**Table:**

*D*—H···*A*	*D*—H	H···*A*	*D*···*A*	*D*—H···*A*
C12—H12*A*···Br1	0.93	3.04	3.866 (4)	148
C2—H2*A*···F1^i^	0.93	2.50	3.081 (4)	121
C5—H5*A*···Br1^ii^	0.93	3.01	3.767 (4)	140
C6—H6*A*···Br1^iii^	0.93	3.05	3.756 (4)	134
C3—H3*A*···Br2^iv^	0.93	2.90	3.810 (4)	166

Symmetry codes: (i) *x*, −*y*+5/2, *z*−1/2; (ii) −*x*, −*y*+1, −*z*; (iii) −*x*, −*y*+2, −*z*; (iv) *x*, −*y*+3/2, *z*−1/2.
